# The tongue features associated with chronic kidney disease

**DOI:** 10.1097/MD.0000000000025037

**Published:** 2021-03-05

**Authors:** Jia-Ming Chen, Ping-Fang Chiu, Feng-Mei Wu, Po-Chi Hsu, Li-Jyun Deng, Chia-Chu Chang, John Y. Chiang, Lun-Chien Lo

**Affiliations:** a Graduate Institute of Chinese Medicine, China Medical University, Taichung; b Department of Traditional Chinese Medicine; c Nephrology Division, Department of Internal Medicine; d Nursing Department, Changhua Christian Hospital, Changhua; e Division of Nephrology, Department of Internal Medicine, Kuang Tien General Hospital, Taichung; f Department of Computer Science and Engineering, National Sun Yat-sen University, Kaohsiung; g School of Chinese Medicine, China Medical University, Taichung; h Department of Chinese Medicine, China Medical University Hospital, Taichung, Taiwan.

**Keywords:** automatic tongue diagnosis system, chronic kidney disease, protocol, tongue examination, traditional Chinese medicine

## Abstract

**Background:**

Traditional Chinese medicine (TCM) tongue diagnosis plays an important role in differentiation of symptoms because the tongue reflects the physiological and pathological condition of the body. The automatic tongue diagnosis system (ATDS), which noninvasively captures tongue images, can provide objective and reliable diagnostic information. Chronic kidney disease (CKD) currently is an important global public health problem and contributor to morbidity and mortality from non-communicable diseases. Thus, it is interesting to analyze and probe the relationship between tongue examination and CKD.

**Methods:**

This protocol is a cross-sectional, case-controlled observational study investigating the usefulness of the ATDS in clinical practice by examining its efficacy as a diagnostic tool for CKD. Volunteers over 20 years old with and without CKD will be enrolled. Tongue images will be captured and the patients divided into 2 groups: CKD group and healthy group. Nine primary tongue features will be extracted and analyzed, including tongue shape, tongue color, tooth mark, tongue fissure, fur color, fur thickness, saliva, ecchymosis, and red dots.

**Result:**

The results of this study will systematically evaluate tongue manifestations of patients and examine its efficacy as an early detection and diagnosis of CKD.

**Discussion:**

The aim of this protocol is to investigate discriminating tongue features to distinguish between CKD and normal people, and establish differentiating index to facilitate the noninvasive detection of CKD.

**Trial registries:**

ClinicalTrials.gov; Identifier: NCT04708743.

## 1 Introduction

Chronic kidney disease (CKD), also called chronic kidney failure, is an important global public health problem. According to related literatures, the prevalence of CKD is about 11%.^[1]^ CKD is described as a sustained reduction in glomerular filtration rate or evidence of structural or functional kidney abnormalities.^[2]^ CKD symptoms include fatigue, persistent itching, peripheral numbness, sleep disturbances, muscle twitches and cramps, swelling of feet and ankles, nausea, and vomiting. Factors that contribute to these symptoms include anemia, uremic toxins, reduced renal capacity, chronic disease-related inflammation, and psychological stress associated with long-term illness.^[3]^ When chronic kidney disease develops into end stage renal disease (ESRD), dialysis or a renal transplant is required to maintain life.^[4]^

The growing prevalence and progression of CKD raises concerns about our capacity to manage its economic burden to patients, caregivers, and society.^[5]^ The societal direct and indirect costs of CKD and ESRD are substantial and increase year by year. ^[6]^ There is significant variability in the evidence about direct and indirect costs attributable to CKD and end-stage renal disease, with the most complete evidence concentrated on direct health care costs of patients with advanced to end-stage CKD. ^[7]^ Therefore, it is essential to find effective and conservative treatment methods to delay the progression of CKD.^[8]^

Diagnosis in TCM is based on 4 procedures, observation, smelling or listening, inquiry, and palpation. Tongue diagnosis, serving as a vital noninvasive tool to provide useful clinical information, plays a pivotal role in TCM.^[9]^ The tongue is considered to reflect the physiological and pathological condition of the body, as well as the degree and progression of disease, through the meridians that connect the tongue to the internal organs.^[10,11]^ By observing tongue features, TCM practitioners can probe qi-blood, yin-yang disorders which are important in treatment selection and prognosis. Clinically, practitioners observe tongue characteristics, such as tongue color and shape, fur color and thickness, and the amount of saliva, to help deduce the primary pattern of a patient. However, tongue diagnosis is often biased by subjective judgment, which originates from personal experience, knowledge, diagnostic skills, thinking patterns, and color perception/interpretation. The inconsistency of subjective diagnosis can be improved by using the development of validated instruments.^[12]^

The automatic tongue diagnosis system (ATDS) has shown high consistency and can provide objective and reliable information and analysis of tongue features, facilitating doctors in making effective observations and diagnoses of specific diseases.^[13]^ Previous studies have been conducted on exploring the association between tongue characteristics and specific diseases, including rheumatoid arthritis,^[14]^ breast cancer,^[15]^ type 2 diabetes,^[11]^ metabolic syndrome,^[16]^ dysmenorrhea,^[17]^ ischemic stroke, ^[18]^ and gastroesophageal reflux disease.^[19]^

However, to the best of our knowledge, no study has yet been performed on the comprehensive scrutiny of tongue features in patients with CKD using ATDS. The objectives of this protocol are to apply the noninvasive ATDS to evaluate tongue manifestations in patients with CKD, and to provide valuable information for clinical doctors, which can be used to facilitate the early detection and diagnosis of CKD, to analyze the current status of patients, and to dynamically schedule treatment plans.

## 2 Methods

### 2.1 Ethics approval and consent to participate

This protocol has been reviewed and approved by the Institutional Review Board of Changhua Christian Hospital, Taiwan (IRB no. 190404) on May 2019. The protocol identification number at https://clinicaltrials.gov is NCT04708743 (registration date: January 14, 2021). This study is conducted in accordance with the principles of the Declaration of Helsinki. Written informed consent will be obtained from all patients before enrollment. Personal information about potential and enrolled participants will be collected, shared, and maintained in an independent closet in order to protect confidentiality before, during, and after the trial.

### 2.2 Participants

We recruited outpatients from the Department of Nephrology and Traditional Chinese Medicine of Changhua Christian Hospital from July 2019. All individuals were recruited were informed of the study purpose, procedures, potential risks and benefits, and then the consent form was signed. Subjects will be eligible if they satisfy the following criteria: age over 20 years old; experimental group with CKD stage 3 to 5 (estimated glomerular filtration rate < 60 ml/minute/1.73^2^) including dialysis patients; control group with not any medication history and visit clinic for health examination; with volunteered to join this research and signed the institutional review board agreement. Subjects with any of the following conditions will be excluded: cancer; acute infection; unable to protrude the tongue stably; risk of temporomandibular joint dislocation.

### 2.3 Study design

This protocol is a cross-sectional, case-controlled observational study investigating the usefulness of the ATDS in clinical practice by examining its efficacy as a diagnostic tool for CKD stage 3 to 5 patients. After giving their consent, participants will undergo tongue image capturing using the ATDS. The ATDS examination will be performed under constant environmental conditions and by the same educated operator. The tongue images of both groups: experimental and control groups, were collected by the validated Automatic Tongue Diagnosis System with the corresponding tongue features automatically extracted. We analyzed the data of the subjects and the tongue features from the ATDS.

### 2.4 Data collection

Information including demography, body mass index (BMI, kg/m^2^), CKD staging, estimated glomerular filtration rate and blood urea nitrogen were gathered for each subject. The duration of CKD history, comorbidity and complication in experimental group were also collected. Tongue images were collected for each subject to further derive the relevant tongue features of every participant. All personal details and photographs of subjects recruited were encrypted to ensure confidentiality.

### 2.5 Intervention: automatic tongue diagnosis system

**Figure 1:**
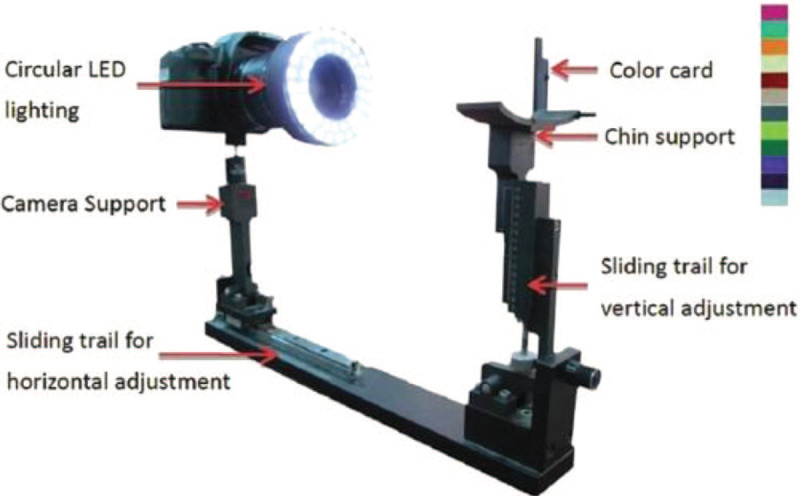
Components of the ATDS.

**Figure 2:**
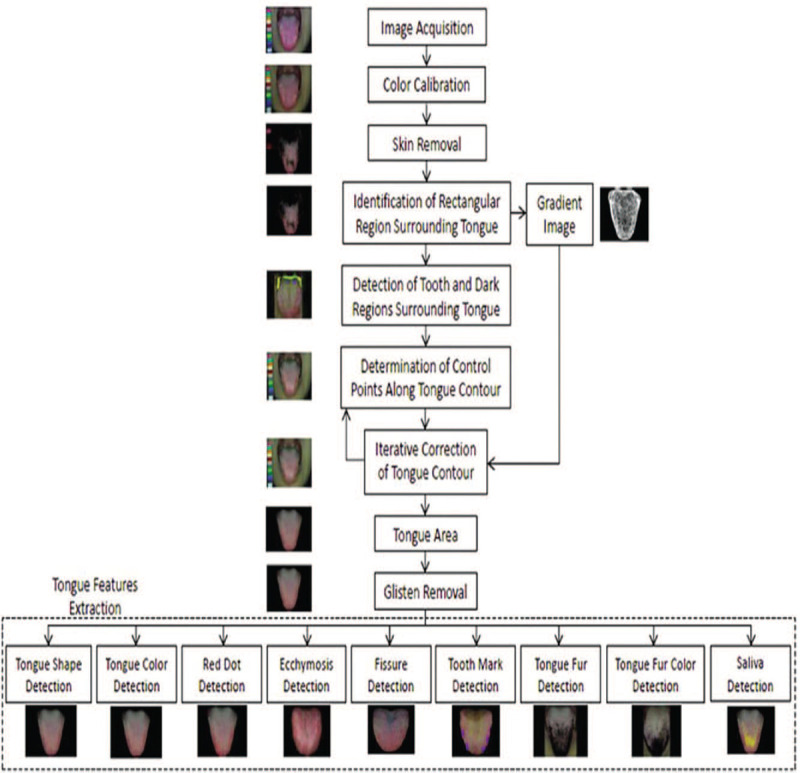
The processing steps of ATDS analysis.

As shown in Figure 1, the ATDS was developed to capture tongue images and automatically extract features reliably to assist the diagnosis of TCM practitioners. The chassis of ATDS is custom-made, while the remaining parts, for example, camera, circular LED light source, etc., can be purchased off-the-shelf. The value of ATDS hinges on its ability to segment the tongue region and extract tongue features automatically. Figure 2 demonstrates the processing steps in 3 major functions, that is, image capturing and color calibration, tongues area segmentation, and tongue feature extraction, embedded in the ATDS.^[13,20]^ There are 9 primary features for TCM clinical tongue diagnosis, namely, tongue color, tongue shape, saliva, tongue fur, tongue quality, tongue fissure, ecchymosis, tooth mark, and red dot. Features extracted are further sub-divided according to the areas located, that is, spleen–stomach, liver–gall-left, liver–gall-right, kidney, and heart–lung areas. The experience of TCM practitioners and multiple professional opinions are integrated to forge the final classification based on the aforementioned characteristics of tongue features derived.

A complete listing of the tongue features extracted is summarized below:

1. Tongue color: Includes slightly white, slightly red, red, dark red, and dark purple
2. Tongue shape: Includes shape (thin and small, moderate, fat and large) and tongue body (normal, tilted to the left, tilted to the right)
3. Saliva: Includes total area and the amount of saliva (none, little, normal, excessive)
4. Tongue fur: Includes fur color (white, yellow, dye), amount, average covering area, maximum covering area, minimum covering area, degree of thickness (none, thin, thick).
5. Tongue quality: Organs corresponding to the covering area (spleen-stomach, liver-gall-left, liver-gall-right, kidney, heart-lung areas)
6. Tongue fissure: Includes amount, average covering area, shortest length, longest length
7. Ecchymosis: Amount, average coving area, maximum covering area, minimum covering area.
8. Tooth mark: Includes number, average covering area, maximum covering area, minimum covering area.
9. Red dot: Includes number, average covering area, maximum covering area, minimum covering area.

### 2.6 Outcome measures

#### 2.6.1 Primary outcome measures

Nine primary tongue features will be extracted from the ATDS as follows: tongue shape: small and thin, moderate, large, and fat; tongue color: slightly white, slightly red, red, dark red, and dark purple; tooth mark: includes number, average covering area, maximum covering area, minimum covering area, and organs corresponding to the covering area; tongue fissure: amount, average covering area, shortest length, and longest length; fur color: white, yellow, and dye; fur thickness: none, thin, thick; fur amount, average covering area, maximum covering area, minimum covering area, and organs corresponding to the covering area; saliva: includes total area and the amount of saliva (none, little, normal, excessive); ecchymosis: amount, average covering area, maximum covering area, minimum covering area, and organs corresponding to the covering area; red dots: number, average covering area, maximum covering area, minimum covering area, and organs corresponding to the covering area. Feature identification will be further subdivided into 5 segments (spleen–stomach, liver-gall-left, liver-gall-right, kidney, and heart–lung area) according to the theory of traditional Chinese medicine.

### 2.7 Sample size

We calculated the sample size will be 453 with power = 0.9, alpha = 0.05, effect size convention *r* = 0.3, and an anticipated drop-out rate of 10%, using G∗Power 3.0.1.0 software.

### 2.8 Data analysis

The tongue features of the subjects participating in the study were extracted by ATDS. All statistical analyses will be performed using the SPSS statistical package program, version 19.0 (SPSS Inc., Chicago, IL). The Mann–Whitney test was performed on the data sets acquired in the experimental and control groups to identify the features with significant difference (*P* < .05). The Mann–Whitney test is a non-parametric test used to compare 2 independent groups of sampled data and without the condition of normal distributions. The test statistic for the Mann–Whitney test is *U*. This value is compared to a table of critical values for *U* based on the sample size of each group.

### 2.9 Data monitoring

Data monitoring committee is not needed because of this observational study.

## 3 Discussion

According to the theory of Traditional Chinese medicine, TCM syndrome differentiate on depends mainly on 4 ways of diagnosis. Among them, observation diagnosis ranks first, and tongue is the major subject of observation. The tongue is thought to be an outer manifestation of the status of the viscera, and can be divided to spleen–stomach area, liver–gall-right area, liver–gall-left area, kidney area, and heart–lung area. It is widely believed that the tongue is connected to the internal organs through meridians; thus, the conditions of organs, qi, blood, and body fluids, as well as the degree and progression of disease are all reflected on the tongue.^[21,22]^ Organ conditions, properties, and variations of pathogens can be found through observation of tongue. For example, changes in the tongue property primarily reflect organ status and the flow of qi and blood; variations in tongue fur can be employed to determine the impact of exogenous pathogenic factors and the flow of stomach qi. In clinical practice of TCM, practitioners observe the characteristics of tongue, such as the color, shape, and the amount of saliva, before deducing the primary ailment of a patient.

There are several studies that discuss the relationship between CKD and tongue characteristics such as tongue coating thickness, tongue coating microbiota, and metabolic markers, and tongue temperature. Pieralisi et al investigated tongue coating frequency and its colonization by yeasts in a group of CKD patients.^[23]^ Anuradha et al analyzed the salivary content of sodium, potassium, calcium, urea, bicarbonate, and oral manifestations in patients with CKD.^[24]^ Lin et al reported that whether there is an association between tongue coating thickness and laboratory, histological variables in idiopathic membranous nephropathy patients.^[25]^ However, the proposed study will provide more details and evidence of the usefulness of tongue image analysis for the identification of CKD. In addition, this method of tongue diagnosis could be utilized in clinical practice and education. We are interested in studying the differences between them, which might be visible on tongue images, and in assessing the relationship between CKD and tongue diagnosis.

In conclusion, the results of this trial are expected to provide valuable evidence supporting the use of tongue diagnosis to evaluate the status of patients with CKD, helping clinical doctors to identify potential problems, and implement proper management of these conditions.

## 4 Trial status

IRB (protocol No.: 190404) was initially approved on August 23, 2019. NCT04708743 was registered on January 14, 2021. Recruitment began in August 2019. Expected date when recruitment will be completed on Mar 31, 2021.

## Acknowledgments

We appreciate the researchers, who have supported us with their helpful suggestions.

## Author contributions

**Conceptualization:** Jia-Ming Chen, Chia-Chu Chang, Lun-Chien Lo.

**Data curation:** Ping-Fang Chiu MD, Feng-Mei Wu.

**Formal analysis:** Lun-Chien Lo.

**Investigation:** Jia-Ming Chen, Lun-Chien Lo.

**Methodology:** John Y Chiang, Po-Chi Hsu.

**Project administration:** Li-Jyun Deng.

**Software:** John Y Chiang.

**Supervision:** Lun-Chien Lo.

**Writing – original draft:** Jia-Ming Chen.

**Writing – review & editing:** Lun-Chien Lo.
